# Serotonin syndrome triggered by postoperative administration of serotonin noradrenaline reuptake inhibitor (SNRI)

**DOI:** 10.1186/s40981-019-0275-5

**Published:** 2019-08-27

**Authors:** Junko Takata, Tomoko Arashi, Ayako Abe, Shoko Arai, Naoko Haruyama

**Affiliations:** Department of Anesthesiology, Tokyo Metropolitan Police Hospital, 4-22-1 Nakano, Nakano-ku, Tokyo, 164-8541 Japan

**Keywords:** Serotonin syndrome, SNRI, Fentanyl, Serotonin

## Abstract

**Background:**

Serotonin syndrome is a rare but potentially severe disease, which is caused by hyperstimulation of serotonin receptors in the central nervous system. Several antidepressants exert their effect by modulating intrasynaptic serotonin concentration and anesthetics may affect the metabolism of serotonin, implicating to induce serotonin syndrome in patients taking those antidepressants.

We present a case which provoked serotonin syndrome immediately after taking serotonin noradrenaline reuptake inhibitor (SNRI) in the postoperative period.

**Case presentation:**

A 31-year-old female underwent laparoscopic ovarian cystectomy under general anesthesia with propofol, fentanyl, and remifentanil. She has been taking duloxetine, a SNRI for depression. She developed myoclonus seizure with an increase of blood pressure and heart rate after taking duloxetine on the day after the surgery, which was subsided by a non-selective serotonin receptor antagonist.

**Conclusions:**

Anesthesiologists should be aware of the risk of perioperative serotonin syndrome in patients taking antidepressants affecting serotonin metabolism.

## Background

Serotonin syndrome is relatively rare but a potentially life-threatening spectrum of drug-induced toxicity [1]. It results from therapeutic drug use, intentional self-poisoning, or inadvertent interactions between drugs, and hyperstimulation of 5-hydroxytryptamine (5-HT) receptors in the central nervous system. Although serotonergic agents, predominantly antidepressants, have been regarded as the major cause of this syndrome [2], opioid analgesics commonly used during anesthesia are also associated with serotonin toxicity [3]. However, little attention is paid to perioperative serotonin syndrome [4]. Here we report a case which developed serotonin syndrome triggered by duloxetine, a serotonin noradrenaline reuptake inhibitor (SNRI) after general anesthesia with propofol, fentanyl, and remifentanil.

## Case presentation

A 31-year-old female (height 165.6 cm, weight 53.1 kg) was scheduled for laparoscopic ovarian cystectomy. Her past medical history was depression, and she has been taking duloxetine, a SNRI 60 mg/day (in the morning) and mirtazapine, a noradrenergic and specific serotonergic antidepressant (NaSSA) 30 mg/day at bedtime for 1 year. Her mental status was stable and these medications was continued until the day of the surgery.

General anesthesia was induced with propofol, remifentanil, rocuronium, and maintained with propofol and remifentanil. Blood pressure and heart rate were stable during anesthesia, and fentanyl 200 μg and flurbiprofen 50 mg along with local infiltration of 0.75% ropivacaine 6.4 ml was administered for postoperative analgesia at the end of the surgery. The duration of the surgery and anesthesia were 80 and 123 min, respectively. She emerged from general anesthesia with no abnormal symptoms. As the surgery was performed in the evening, she skipped to take mirtazapine on the day of the surgery.

One day after the surgery, the patient took the usual dose of duloxetine 60 mg in the morning. Three to 5 min later, she claimed hot flush, stiffness around her neck and shoulders, and muscle rigidity in the masseter. The blood pressure was 112/78 mmHg, and the heart rate was 77 beats/min at this time. Medical examination showed neuromuscular abnormalities (muscle rigidity, myoclonus, stiff neck, tremor, and hyperreflexia) and autonomic instability (diaphoresis, tachypnea, tachycardia, and mydriasis). Myoclonus seizure had appeared intermittently and repeated mainly on the upper extremities and neck. During the myoclonus seizure, her heart rate and systolic blood pressure had raised to 140 beats/min and over 160 mmHg, respectively. The respiratory rate also raised to 28–35/min. She was febrile (37.4 °C) and complained of anxiety due to the situation occurring to her. Given the neuromuscular abnormalities and recent surgery, malignant syndrome and neuroleptic malignant hyperthermia were assumed and initial dose of dantrolene 40 mg was given immediately, and body cooling was started as well. Blood test revealed no abnormalities including creatine kinase level, thyroid hormone, glucose, and electrolytes. Dantrolene was not effective and anesthesiologists were consulted from the gynecologists for further treatment.

A diagnosis of serotonin syndrome was made based on the findings that the onset of the symptoms occurred immediately after taking SNRI; symptoms such as clonus, agitation, hypertension, and tachycardia corresponded to the diagnostic criteria (Hunter criteria; Table 1) [2]. Duloxetine and mirtazapine were stopped. Serum levels of serotonin was 4 (normal range 57–230) ng/ml, dopamine 21 (<20) pg/ml, noradrenaline 434 (100–450) pg/ml, and adrenaline 294 (<100) pg/ml. Myoclonus seizures occurred repeatedly, tachycardia, tachypnea, and hypertension persisted, and the patient was admitted to the intensive care unit.

**Table 1:** Hunter criteria

Hunter criteria	
History of serotonergic agent usage At least one or more of the followings: 1. Clonus: spontaneous, inducible, ocular 2. Agitation 3. Autonomic dysfunction (i.e., hyperthermia) 4. Tremor 5. Hyperreflexia	

Clonazepam 1.0 mg (per oral) was given orally for anxiety and hyperexcitatory symptoms, and intravenous landiolol 0.01 mg/kg/min was started to control heart rate. Non-selective serotonin receptor antagonist, cyproheptadine (per oral, 12 mg), was also initiated to decrease intrasynaptic serotonin level through 5-HT blocking effect. Myoclonus had disappeared soon after administration of cyproheptadine and hemodynamic had stabilized. Intralipid (20%) 100 ml was also administrated for its transient ability to draw tissue and receptor bound serotonergic drugs into lipid-expanded intravascular compartment and trap them into a lipid phase. Additional cyproheptadine 4 mg was given 6 h later and 14 h later from its first administration to continue its antagonistic effect. Two days after surgery, the patient was transferred to the general ward. Serum serotonin effect site concentration was 3 ng/ml. Four days after the surgery, the patient was discharged from the hospital and advised to consult her psychiatrist for the further use of SNRI and NaSSA. (Timeline of development of symptoms and management is presented in Table 2).

**Table 2:** Timeline of development of symptoms and management

Time	Symptoms	Management
Day 1 8:33 a.m.	After taking SNRI, hot flush, stiffness around the neck and shoulder, diaphoresis, and muscle rigidity in the masseter appeared. BP 112/78 mmHg, 77 bpm, BT 37.4 °C	Body cooling Intravenous administration of dantrolene 40 mg for muscle rigidity. No abnormalities were found in the blood test.
10:10	No raise in serum serotonin level (4 ng/ml)	Diagnosed as serotonin syndrome Serotonergic agents (duloxetine and mirtazapine) were stopped immediately.
12:45	Myoclonus and tremor on the upper extremities. Tachycardia (120 bpm) and diaphoresis	
13:00	Myoclonic seizures on the upper extremities and neck. Tachycardia (140 bpm), BP 162/102 mmHg, respiratory rate 28/min	
13:20	Myoclonic seizures on the shoulders and neck. Tachycardia (140 bpm), BP 169/120 mmHg, respiratory rate 35/min	Admitted to the intensive care unit. Clonazepam 1.0 mg (per oral), intralipid (20%) 100 ml, and started landiolol 0.01 mg/kg/min
16:10	No myoclonus was seen after the initial dose of cyproheptadine. Vital was stabilized soon.	Cyproheptadine 12 mg (per oral)
22:00		Cyproheptadine 4 mg (per oral)
Day 2 6:00		Cyproheptadine 4 mg (per oral) Moved to general ward
Day 4		Discharged from the hospital

## Discussion

Serotonin syndrome is not widely recognized among the anesthesiologists compared to malignant hyperthermia (MH) and neuroleptic malignant syndrome (NMS). Its variable and overlapping manifestations make it difficult to diagnose. Differential diagnosis includes MH, NMS, and anticholinergic syndrome; all of which may present with hyperthermia and neuromuscular abnormalities. In this case, it was relatively easy to rule out MH because we managed the anesthesia by total intravenous anesthesia (TIVA) and did not use any agents which may trigger malignant hyperthermia, such as inhaled halogenated anesthetics and depolarizing muscle relaxants. As the patient was taking antipsychotic medications, NMS was also considered, but it was excluded after careful physical examination. Clinical condition of NMS is dopamine antagonism and it is characteristic with extrapyramidal symptoms, lead pipe rigidity, bradykinesia, and a history of dopamine antagonist use. The onset of NMS is known to be relatively slow [1].

In this case, serotonin syndrome had provoked just after the patient took the usual dose of SNRI at the postoperative period. SNRI alone has the potential risk of provoking serotonin syndrome and attention must be paid, especially when the dose is increased. Considering the fact that the patient’s mental status was stable with the same dose of duloxetine for a long period, and she had taken the usual dose of duloxetine in the morning at the surgery day, other additive cause which may modulate serotonin system was suspected. Fentanyl is a widely used synthetic phenylpiperidine opioid and it also functions as a 5-HT_1A_ agonist by increasing serotonin release [5]. It also has a weak serotonin reuptake inhibition effect which concomitantly increases intrasynaptic serotonin levels [6]. The last fentanyl use was about 15 h before the patient got into serotonin syndrome, and considering its short half-time, it is uncertain whether the fentanyl used during the operation had contributed to build serotonin syndrome. But it was the only serotonergic agents used perioperatively besides the patient’s usual medicine and might have had some interaction. Some cases are reported regarding the interaction between fentanyl and serotonin selective reuptake inhibitor (SSRI) [4]. As we do not have any direct evidence, re-administration of SNRI alone could be the more likely cause of serotonin syndrome onset in this case.

Symptoms of serotonin syndrome have a direct correlation with intrasynaptic serotonin level, but the blood serotonin level does not reflect the severity of the disease [2]. Serum serotonin concentration was measured in this case at day 1 and day 2, which was quite low and did not correlate with the symptoms. As the diagnosis of serotonin syndrome is made by objective symptoms and no specific laboratory test exits, it is important for the anesthesiologist to have the knowledge of serotonin syndrome. Drugs associated with serotonin syndrome include opioid analgesics, fentanyl, pethidine, and tramadol those of which anesthesiologist frequently use in the perioperative period [3].

The first step management of serotonin syndrome is the discontinuation of offending serotonergic agents. Majority of the cases are reported to have been resolved within 24 h after discontinuation of serotonergic agents and supportive care [1, 7]. However, some cases require more intense care. Symptoms of severe serotonin syndrome includes rhabdomyolysis, renal failure, severe metabolic acidosis, and disseminated intravascular coagulation. Moderate to severe serotonin syndrome is said to require serotonin receptor antagonists [1]. It was characteristic in this case that the symptoms of serotonin syndrome subsided shortly after oral administration of cyproheptadine.

## Conclusion

We experienced the case of a 31-year-old female who developed serotonin syndrome after general anesthesia. Interaction between intraoperative use of fentanyl and postoperative intake of SNRI was not sure in this case, but re-administration of SNRI could be the cause of developing serotonin syndrome. Anesthesiologists need to be more aware of the risk that serotonergic agents, predominantly antidepressants, could trigger perioperative serotonin syndrome.

## Data Availability

The datasets generated and analyzed during this case report are available from the corresponding author on reasonable request.

## Abbreviations

5-HT: 5-Hydroxytryptamine
MH: Malignant hyperthermia
NaSSA: Noradrenergic and specific serotonergic antidepressant
NMS: Neuroleptic malignant syndrome
SNRI: Serotonin noradrenaline reuptake inhibitor
SSRI: Serotonin selective reuptake inhibitor
TIVA: Total intravenous anesthesia
